# Epidemiologic features and management of hypertension in Tunisia, the results from the Hypertension National Registry (NaTuRe HTN)

**DOI:** 10.1186/s12872-022-02584-y

**Published:** 2022-03-29

**Authors:** Leila Abid, Rania Hammami, Ikram Chamtouri, Meriam Drissa, Selim Boudiche, MohamedAmine Bahloul, Hedi BenSlima, Khaled Sayahi, Selma Charfeddine, Emna Allouche, Lamia Rais, Badr Kaab, Hassen IbnHadjamor, Lilia BenFatma, Riadh Garbaa, Sabrine Boukhris, Manel Ben Halima, Nesrine Amdouni, Chaima Ghorbel, Sabrine Soudani, Imen Khaled, Syrine Triki, Feten Bouazizi, Imen Jemai, Ouday Abdeljalil, Yemna Ammar, Amani Farah, Adnen Neji, Zeineb Oumayma, Sana Seghaier, Samir Mokrani, Hamza Thawaba, Hela Sarray, Khalil Ouaghlani, Houssem Thabet, Zeineb Mnif, Fatma Boujelban, Mohamed Sghaier, Roueida Khalifa, Sami Fourati, Yasmin Kammoun, Syrine Abid, Chihab Hamza, Syrine Ben Jeddou, Lassaad Sabbah, Rim Lakhdhar, Najla Dammak, Tarek Sellami, Basma Herbegue, Alia Koubaa, Faten Triki, Tarek Ellouz, Aicha Hmoudi, Ikhlas BenAmeur, MohamedMongi Boukhchina, Neila Abid, Wejdene Wachtati, Nizar Nasrallah, Yousra Houidi, Fathia Meghaieth, Elhem Ghodhbane, Mounira Chayeb, Sarra Chenik, Samira Kaabachi, Nizar Saadaoui, Ines BenAmeur, Moufida Affes, Sana Ouali, Mouna Chaker, Hela Naanaa, Meriem Dghim, Mourad Jarrar, Jihene Mnif, Ahmed Turki, Ihsen Zairi, Jamel Langar, Safa Dardouri, Imen Hchaichi, Rafik Chettaoui, Wajih Essmat, Amel Chakroun, Khadija Mzoughi, Rachid Mechmeche, Afef BenHalima, Sahar BenKhala, Slim Chtourou, Abdelkader Maalej, Mohsen Ayari, Moufid Hadrich, Rami Tlili, Fares Azaiez, Imen Bouhlel, Samira Sahnoun, Habib Jerbi, Imtinene BenMrad, Leila Riahi, Mohamed Sahnoun, Abdelhamid BenJemaa, Amel BenSalem, Bassem Rekik, Maroua BenDoudou, Rachid Boujneh, Anissa Joulak, Yosra Mejdoub, Imen Gtif, Gouider Jribi, Elyes Naffeti, Habib Gamra, Soraya BenYousef, Wissem Sdiri, Najeh BenHalima, Youssef BenAmeur, Salem Kachboura, Sondes Kraiem, Wafa Fehri, Leila Bazdeh, MohamedSami Mourali, Sami Milouchi, Habiba Drissa, Faouzi Maatouk, Lilia Zakhama, Faouzi Addad, Samir Kammoun, Salem Abdesselem

**Affiliations:** 1 Tunisian Society of Cardiology and Cardiovascular Surgery, Tunis, Tunisia; 2 Cardiology Department, Hedi Chaker-Sfax University Hospital, Sfax, Tunisia; 3 Cardiology Department B, Fattouma Bourguiba University Hospital, Monastir, Tunisia; 4 Cardiology Department, La Rabta 2 (Pr Drissa) University Hospital, Tunis, Tunisia; 5 Cardiology Department, La Rabta 1 (Pr Mourali) University Hospital, Tunis, Tunisia; 6 Cardiology Department, Hospital of Menzel Bourguiba, Bizerte, Tunisia; 7 Cardiology Department, ElKef Hospital, Elkef, Tunisia; 8 Cardiology Department, Charles Nicole University Hospital, Tunis, Tunisia; 9 Nephrology Department, La Rabta University Hospital, Tunis, Tunisia; 10 Cardiology Department, Tahar Sfar Hospital, Mahdia, Tunisia; 11 Private Sector, Medenine, Tunisia; 12 Cardiology Department, Habib Bourguiba Hospital, Medenine, Tunisia; 13 CSB, Medenine, Tunisia; 14 Habib Bourguiba Hospital, Medenine, Tunisia; 15 Hospital of Tozeur, Tozeur, Tunisia; 16 Private Sector, Tunis, Tunisia; 17 Hospital of Mateur, Bizerte, Tunisia; 18 Private Sector, Mahdia, Tunisia; 19 Cardiology Department, Farhat Hached Hospital, Sousse, Tunisia; 20 NSSF, Sfax, Tunisia; 21 Private Sector, Sfax, Tunisia; 22 Private Sector, Ben Arous, Tunisia; 23 Private Sector, Tunis, Tunisia; 24 Private Sector, Sfax, Tunisia; 25 Private Sector, Ariana, Tunisia; 26 Nephrology Department, Hedi Chaker-Sfax University Hospital, Sfax, Tunisia; 27 Private Sector, Kebeli, Tunisia; 28 CSB, Ben Arous, Tunisia; 29 CSB, Tataouine, Tunisia; 30 Private Sector, Gabes, Tunisia; 31 Private Sector, Ariana, Tunisia; 32 CSB, Tunis, Tunisia; 33 Cardiology Department, The Main Military Instruction Hospital of Tunis, Tunis, Tunisia; 34 La Rabta 2 (Pr Drissa) University Hospital, Tunis, Tunisia; 35 Private Sector, Nabeul, Tunisia; 36 Private Sector, Sousse, Tunisia; 37 Cardiology Department, Habib Thameur Hospital, Tunis, Tunisia; 38 CSB, Gabes, Tunisia; 39 Private Sector, Gafsa, Tunisia; 40 Hospital of Habib Bougatfa, Bizerte, Tunisia; 41 Cardiology Department, Abderrahmen Mami-Ariana Hospital, Ariana, Tunisia; 42 Cardiology Department, Mahres Hospital, Sfax, Tunisia; 43 Private Sector, Nabeul, Tunisia; 44 Cardiology Department, Mongi Slim Hospital, Tunis, Tunisia; 45 Private Sector, Sousse, Tunisia; 46 Community Medicine Department, Hedi Chaker-Sfax University Hospital, Sfax, Tunisia; 47 Laboratory of Screening Cellular and Molecular Process, Centre of Biotechnology of Sfax, Sfax, Tunisia; 48 Cardiology Department, University Hospital Sahloul, Sousse, Tunisia; 49 Cardiology Department A, Fatouma Bourguiba Hospital, Monastir, Tunisia; 50 Cardiology Department, Internal Security Forces Hospital, Tunis, Tunisia; 51 Cardiology Department, Bougatfa Hospital, Bizerte, Tunisia; 52 Cardiology Department, Ibn El Jazzar Hospital, Kairouan, Tunisia

**Keywords:** Hypertension, Blood pressure control, Epidemiology, Predictors, Registry

## Abstract

**Background:**

Hypertension is the leading cause of morbi-mortality in our country. Thus, we conducted this national survey on hypertension to analyze the profile of the Tunisian hypertensive patient and to assess the level of blood pressure control.

**Methods:**

Nature HTN is an observational multicentric survey, including hypertensive individuals and consulting their doctors during the period of the study. Blood pressure measurements were conducted during consultation, using a standardized auscultatory or oscillometric sphygmomanometer after at least 15 min of rest. The diagnosis of new hypertension is based on the 2018 ESC/ESH criteria. The primary endpoint of our study was uncontrolled hypertension defined by a systolic blood pressure ≥ 140 mmHg and/or diastolic blood pressure ≥ 90 mmHg.

**Results:**

Three hundred twenty-one investigators participated in the study. We enrolled 25,890 patients with a female predominance (Sex ratio, 1.21) and an average age of 64.4 ± 12.2 years. Most individuals were treated in the public sector (74%), 39.4% of patients were diabetic, 25.8% were obese, 44.6% were overweight and 14% were smokers. Hypertension was controlled in 51.7% of cases if we consider 140/90 as a BP target, and only in 18.6% if we consider 130/80 as a target. The independent predictors of uncontrolled blood pressure were male sex (OR = 1.09, 95%CI [1.02–1.16]), age > 65 year-old (OR = 1.07, 95% CI[1.01–1.13], diabetes (OR = 1.18, 95% CI [1.11–1.25], Smoking (OR = 1.15, 95% CI [1.05–1.25]), Obesity (OR = 1.14, 95% CI[1.07–1.21]), management in public sector (OR = 1.25, 95% CI [1.16–1.34]), and Heart rate > 80 bpm (OR = 1.59, 95% CI [1.48–1.71]). Contrarily, high educational level (OR = 0.9, 95% CI [0.84–0.97], absence of history of coronary disease (OR = 0.86, 95% CI [0.8–0.93]), salt restriction (OR = 0.48, 95% CI [0.45–0.51]), drug compliance (OR = 0.57, 95% CI[0.52–0.61]), and regular physical activity (OR = 0.77, 95% CI[0.71–0.84]) are strong predictors of blood pressure control.

**Conclusion:**

NaTuRe HTN showed that blood pressure control was reached in more than half of the Tunisian people. The control remains low in patients with high cardiovascular profiles and in those treated in the public sector. A national health program based on therapeutic education, regular control and continuous support to the public institutions is needed to decrease the burden of hypertension incidence rate.

## Background

Hypertension is the most common chronic disease in the world with a prevalence ranging between 30 and 50%. It is considered to be the leading cause of morbi-mortality in adults, especially in low- and middle-income countries. Treatment rates were at most 40% and control rates were less than 25% in most countries in all age and sex groups [1–9]. The prevalence is higher in the elderly and exceeds 60% in people aged > 60 years [3]. Given the widespread of sedentary lifestyles and obesity, the prevalence of hypertension worldwide will continue to rise. It is estimated that the number of hypertensive people will increase by 15–20% by 2025, reaching close to 1.5 billion [2]. Several recent epidemiological studies demonstrated that high blood pressure is under-diagnosed in the 5 continents of the world and despite the development of the therapeutic arsenal, the control of hypertension does not seem to improve in most countries [4, 6, 10–13]. In Tunisia, a middle-income developing country, the latest epidemiological data related to hypertension date back to 2012, from the national survey “TAHINA Study” [14]. The lifestyle of Tunisian people has widely changed in the last years with an increase of sedentariness, overweight and obesity, diabetes, and dyslipidemia… Tunisia is considered as a high cardiovascular risk country [15] and the World Health Organization, as well as other international organizations, estimate that the Tunisian epidemiologic situation will worsen in the next years [16]. The main reason why we envisioned the need for a national multi-centric survey is to analyze the epidemiologic profile of hypertension in Tunisia and to assess the level of blood pressure control as well as the predictors of uncontrolled hypertension.

## Patients and methods

NaTuRe HTN registry is an observational multi-centric national study, conducted in all the governorates of Tunisia, in both public and private health sectors. Patients were included between 15 April 2019 and 15 May 2019 (Ramadan in between). Different investigators ensured the enrollment and the clinical examination: Cardiologists, General Practitioners as well other specialists such as Nephrologists, Endocrinologists, and Internists. We included all patients with known or newly diagnosed hypertension who consulted their doctors during the enrollment period.

### Inclusion criteria

During the office visits, we included patients with a history of or newly diagnosed elevated blood pressure and older than 18 years, after signing a consent form.

High blood pressure was confirmed by clinic blood pressure measurements in all patients included in the analysis. Except when hypertension is severe (e.g. grade 3, and especially in high-risk patients), the diagnosis of new hypertension was confirmed according to the ESC/ESH guidelines as either Out-of-office Blood Pressure (BP) measurement above the recommended thresholds or repeated office BP measurements on more than one visit above 140 mmHg for the systolic pressure and/or 90 mmHg for the diastolic pressure [17].

### Exclusion criteria

We excluded from the study patients undergoing hemodialysis, pregnant women, those classified as white coat hypertensive patients, and those who refused to sign the consent form.

### Clinical evaluation and data collection

During the office visit, the physician had to complete the case report form of the registry after the patient’s interrogation and examination.

Information on socio-demographic characteristics including age, gender, education level, health insurance, smoking, diabetes, pulmonary diseases, hypothyroidism, moderate renal failure history (defined by an MDRD creatinine clearance < 60 ml / min [18]), coronary disease as well as the history of stroke was collected.

The interview included questions related to drug compliance and salt intake as well as sport practice. Physical activity was considered as regular when it was performed for at least 30 min three times a week [17].

On physical examination, we measured weight and height to assess body mass index (BMI = weight/height^2^). Obesity is operationally defined as a BMI exceeding 30 kg/m^2^ and is subclassified into moderate (BMI:30–34.9), morbid (BMI:35–39.9), and severe (BMI ≥ 40) [19]. Blood pressure measurements were conducted using a standardized auscultatory or oscillometric sphygmomanometer after at least 15 min of rest. Two separate readings were taken at least three minutes apart and we considered the average of the two measurements. In patients with asymmetric blood pressure between the two arms (difference ≥ 20 mmHg for systolic pressure and 10 mmHg for diastolic pressure), we considered the higher pressure.

We checked on electrocardiogram whether the patient had a sinus rhythm or atrial fibrillation and we searched for left ventricle hypertrophy (LVH) based on the definition recommended by the ESC/ESH guidelines (Sokolow–Lyon index > 35 mm, or R in aVL > _11 mm) [17]. We also searched for LVH on echocardiographic findings (if the patient underwent echocardiography during the last year).

We also noted the last biology tests, performed during the last year before the office visit, especially creatinine, glycaemia, cholesterol, and kaliemia as well as microalbuminuria (we considered that test is valid if performed during the last year for microalbuminuria and within the last six months for the other tests).

To assess blood pressure control, we evaluated only patients with diagnosed hypertension for more than 6 months. The primary endpoint in our study was the rate of hypertension control.

Uncontrolled hypertension was defined according to the ESC/ESH guidelines as average systolic blood pressure (SBP) above 140 mmHg and/or average diastolic blood pressure (DBP) above 90 mmHg [17].

The secondary endpoints were the assessment of the profile of patients with hypertension in Tunisia (age, sex, and cardiovascular factors).

Validation of the study protocol and the consent form by a national ethics committee was also obtained.

The data collected were managed by the Clinical Suite platform (Dacima Software), which complies with international standards including US Food and Drug Administration 21 Code of Federal Regulations Part 11, US Health Insurance Portability and Accountability Act, International Conference on Harmonisation, and Medical Dictionary for Regulatory Activities. The Clinical Suite platform allowed us to track the data entered and to check for inconsistencies and missing data. A steering committee was set up to monitor patient inclusions, verify data sources, perform the audit trail, and prepare the statistical analysis plan for the study.

We confirm that all methods were carried out in accordance with relevant guidelines and regulations. All experimental protocols were approved by the ethics committee of the Hospital of the Internal Security Forces. Informed written consent was obtained from all subjects. In the case of illiterate participants, informed consent was obtained from legal guardians.

### Statistical analysis

All statistical analyses were achieved using the SPSS 23.0 (SPSS, Chicago, IL, USA) statistical package. Continuous variables were presented as means value ± standard deviation in case of Gaussian distribution and as medians as well as extreme values in case of non-Gaussian distribution.

Among patients with old hypertension for more than 6 months, we distinguished two groups according to the hypertension control (controlled group versus uncontrolled group). The comparison between the two groups was achieved by Student’s t-test and Chi2 test for continuous variables and categorical variables, respectively. Univariate logistic regression analyses were used to determine the crude odds ratio with the 95% approximate confidence intervals as estimators of the non-control of hypertension for various characteristics of the study population. To assess the predictors of hypertension non-control, we performed a multivariate logistic regression model. The significance threshold was set at p < 0.05.

The factors associated with uncontrolled pressure were studied by calculating unadjusted (in univariate analysis) and adjusted (ORa) Odds Ratios after multivariate analysis using binary logistic regression. We retained a risk of error of 20% to include the indicator variables in the multivariate analysis.

## Results

The NaTuRe HTN registry concerned 25,890 hypertensive patients, enrolled by 321 investigators from all the Tunisian governorates. The case report form was completed by Cardiologists in 71% of patients, a general practitioner in 25%, and other specialists in 4%. The patients were managed in the public and private sectors but the majority of patients were followed in public centers (78%). The hypertension was newly diagnosed in 2286 patients (8.8%) and in the medical history/record of more than six months in 23,601 persons.

The majority of the patients (16,565, 64%) were included during Ramadan, especially for the private sector.

The epidemiologic and clinical characteristics of the overall population are summarized in Table 1.

**Table 1:** Epidemiologic and clinic chracteristics of the population according to the sex and the management sector

	All population	Female	Male	p (male vs female)	Public	Private	p (public vs private)
N	25,890	14,166	11,700		20,192	5698	
*Age (years)*							
Median (min–max) Mean ± SD	64.00 (18–118) 64.4 ± 12.2	64 (18–118) 64.5 ± 12.2	64 (18–108) 64.2 ± 12.2	0.059*	64 (18–118) 64.46 ± 12.2	65 (18–101) 64.19 ± 12.1	0.4
Age > 65 yo (%)	12,662 (49.3)	6982 (49.7)	5673 (48.8)	0.1	9860 (49.1)	2802 (50.1)	0.4
Ramadan Inclusion (%)	16,565 (64)	9155 (64.6)	7403 (63.3)	0.02	13,173 (35.2)	3392 (59.5)	** < 10**^**–3**^
*Education level*							
Illiterate (%) Primary school (%) Secondary school (%) University school (%) Unspecified (%)	5527 (21.3) 5475 (21.1) 4493 (17.4) 2428 (9.4) 7967 (30.8)	4261 (30.1) 3124 (22.1) 1880 (13.3) 825 (5.8) 4076 (28.8)	1262 (10.8) 2346 (20.1) 2612 (22.3) 1600 (13.7) 3880 (33.2)	**< 10**^**–3**^	4527 (22.4) 4384 (21.7) 3139 (15.5) 1439 (7.1) 6703 (33.2)	1000 (17.6) 1091 (19.1) 1354 (23.8) 989 (17.4) 1264 (22.2)	** < 10**^**–3**^
*Health insurance coverage*							
Private insurance (%) National insurance (%) State Medicare (%) None insurance (%) Unspecified (%)	762 (2.9) 18,453 (71.3) 5052 (19.5) 790 (3.1) 833 (3.2)	399 (2.8) 9806 (69.2) 3114 (22) 408 (2.9) 439 (3.1)	363 (3.1) 8632 (73.8) 1936 (16.5) 382 (3.3) 387 (3.3)	< 10^–3^	434 (2.1) 14,165 (70.2) 4724 (23.4) 520 (2.6) 346 (1.7)	328 (5.8) 4285 (75.2) 328 (5.8) 270 (4.7) 487 (8.5)	< 10^–3^
Smoking (%)	3630 (14)	391 (2.8)	3236 (27.7)	< 10^–3^	3021 (15)	609 (10.7)	< 10^–3^
Diabetes (%)	10,204 (39.4)	5621 (39.7)	4583 (39.2)	0.4	8185 (40.5)	2029 (35.6)	< 10^–3^
Obesity (%)	6979 (25.8)	4927 (34.7)	2065 (17.6)	< 10^–3^	5207 ( 25.9)	2590 (46.3)	< 10^–3^
*Corpulence*							
Overweight (%) Moderate obesity (%) morbid Obesity (%) severe Obesity (%)	11,539 (44.6) 5182 (20) 461 (1.8) 1354 (5.2)	5984 (42.2) 3509 (24.8) 375 (2.6) 1043 (7.4)	5546 (47.4) 1671 (14.3) 85 (0.7) 310 (2.6)	< 10–3	9454 (46.8) 3946 (19.5) 302 (1.5) 959 (4.7)	2085 (36.6) 1236 (21.7) 959 (2.8) 395 (6.9)	< 10^–3^
*BMI (kg/cm*^*2*^*)*							
Median (min–max) Mean ± SD	27.5 (13.7–72.2) 28.1 ± 4.57	28.3 (14–70) 29.01 ± 4.57	27.07 ± 3.93	< 10^–3^*	27.3 (14–72) 27.9 ± 4.3	29 (13.7–66.9) 29.08 ± 5.13	< 10^–3^*
Newly diagnosed HTN (%)	1475 (5.7)	705 (5.1)	770 (6.8)	< 10^–3^	953 (4.7)	522 (9.2)	< 10^–3^
Moderate Renal Failure (%)	1524 (5.9)	672 (4.7)	851 (7.3)	< 10^–3^	1305 (6.5)	219 (3.8)	< 10^–3^
Hypothyroidism (%)	1384 (5.3)	1136 (8)	248 (2.1)	< 10^–3^	1034 (5.1)	350 (6.1)	0.002
Apnea syndrome Confirmed (%) Suspected (%)	469 (1.8) 843 (3.3)	257 (1.8) 499 (3.5)	211 (1.8) 343 (2.9)	0.02	276 (1.4) 505 (2.5)	193 (3.4) 338 (5.9)	< 10^–3^
COPD (%)	519 (2)	137 (1)	382 (3.3)	< 10^–3^	380 (1.9)	139 (2.4)	0.008
Stroke history (%)	1707 (6.6)	796 (5.6)	910 (7.8)	< 10^–3^	1311 (6.5)	396 (6.9)	0.2
Coronary disease (%)	4797 (18.5)	1742 (12.3)	3048 (26.1)	< 10^–3^	3941 (19.8)	856 (15)	< 10^–3^
*SBP (mmHg)*							
Median ( min–max) Mean ± SD	135 (80–260) 138.8 ± 19.6	135 (80–260) 138.5 ± 19.7	140 (85–250) 139.2 ± 19.4	< 10^–3^*	140 (80–260) 138.9 ± 19.7	134.5 (80–260) 138.6 ± 19	0.1*
*DBP (mmHg)*							
Median ( min–max) Mean ± SD	80 (40–140) 79.03 ± 11.1	80 (40–140) 78.8 ± 10.9	80 (40–140) 79.3 ± 11.3	0.005*	80 (40–140) 78.9 ± 11.2	80 (40–140) 79.4 ± 10.7	0.6*
HTN History > 6 months	23,601 (91.1)	13,058 (55.3)	10,543 (44.7)	< 10^–3^	18,613 (95.1)	4988 (90.5)	< 10^–3^
Controlled HTN (Target < 140/90 mmHg) (%)	12,206 (51.7)	6825 (52.4)	5371 (50.9)	0.032	9361 (50.3)	2845 (57)	< 10^–3^
Controlled HTN (Target < 130/80 mmHg) (%)	4386 (18.6%)	2486 (19.1)	1896 (18)	0.033	3357 (18)	1029 (20.6)	< 10^–3^
Heart rate (bpm) Median ( min–max) Mean ± SD	74 (40–150) 73.8 ± 11	74 (40–150) 74.1 ± 11.1	73 (40–150) 73.5 ± 10.9	< 10^–3^*	74 (40–150) 74.3 ± 10.8	70 (40–150) 72.2 ± 11.8	< 10^–3^
Heart rate > 80 bpm (%)	4878 (19.2)	2754 (19.8)	2120 (18.5)	0.009	3895 (19.6)	983 (17.8)	0.003
LVH on EKG or TTE (%)	3377 (13)	1399 (9.9)	1482 (12.7)	< 10^–3^	2125 (10.5)	758 (13.3)	< 10^–3^
Atrial fibrillation (%)	1914 (7.4)	1130 (8)	783 (6.7)	< 10^–3^	1527 (7.6)	387 (6.8)	0.05
Recent lab tests < 6 months	14,737 (56.9)	8202 (57.9)	6521 (55.7)	< 10^–3^	10,302 (77.8)	4435 (51)	< 10^–3^
*Fasting Glucose (g/l)*							
Median ( min–max) Mean ± SD	1.1 (0.5–3.9) 1.3 ± 0.5	1.15 (0.6–3.97) 1.8 ± 6.1	1.1 (0.6–3.9) 1.6 ± 3.7	0.8*	1.129 (0.6–3.9) 1.6 ± 2.5	1.1 (0.6–3.8) 1.9 ± 7.6	< 10^–3^*
*Creatinine (µmol/l)*							
Median ( min–max) Mean ± SD)	81 (30–700) 92.6 ± 50.7	76 (30–681) 85 ± 45.5	89 (32–700) 102 ± 55.11	< 10^–3^*	80 (30–700) 93.1 ± 53.7	85 (30–663) 90.5 ± 38.5	< 10^–3^*
*Creatinine clearance (ml/min)*							
Median (min–max) Mean ± SD	88.4 (7–287) 90.4 ± 33.5	96.4 (7.62–287.3) 98.5 ± 35.3	79.7 (7.3–246) 80.3 ± 28	< 10^–3^*	89.2 (7.3–287) 91 ± 34.6	85 (7.8–262) 88 ± 29	< 10^–3^*
*Total Cholesterol (g/l)*							
Median (min–max) Mean ± SD	1.8 (1–5) 1.9 ± 0.6	1.8 (1–5) 1.96 ± 0.6	1.7 (1–5) 1.84 ± 0.6	< 10^–3^*	1.8 (1–5) 1.8 ± 0.4	1.8 (1–5) 1.9 ± 0.7	0.6*
*K+ (mmol/l)*							
Median (min–max) Mean ± SD	**4.1 (2.4–7)** **4.1** ± 0.4	4.1 (2.5–6.8) 4.15 ± 0.4	4.1 (2.4–7) 4.1 ± 0.4	< 10^–3^*	4.1 (2.45–7) 4.1 ± 0.48	4.1 (2.5–6.8) 4.1 ± 0.42	0.005
Microalbuminuria test during the last year (%)	4042 (15.6)	2226 (15.7)	1814 (15.5)	0.6	2190 (10.8)	1852 (32.5)	< 10^–3^
Positive Microalbuminuria (% among people who got the test)	**1365 (33.8)**	652 (29.3)	713 (39.3)	< 10^–3^*	910 (41.6)	455 (24.61)	< 10^–3^
*Drug treatment*							
No drug (%) Monotherapy (%) Biotherapy (%) tritherapy or more (%)	4037 (15.6) 12,042 (46.5) 6614 (25.5) 3197 (12.3)	2170 (15.3) 6626 (46.8) 3646 (25.7) 1724 (12.2)	1862 (15.9) 5408 (46.2) 2961 (25.3) 1469 (12.6)	0.3	3032 (15) 9794 (48.5) 5032 (24.9) 2334 (11.6)	1005 (17.6) 2248 (39.5) 1582 (27.8) 863 (15.1)	< 10^–3^
*Antihypertensive drugs*							
ACE (%)	11,770 (45.5)	6420 (45.3)	5341 (45.6)	0.5	9251 (45.8)	2519 (44.2)	0.03
ARB (%)	5587 (20.6)	3012 (21.3)	2569 (22)	0.17	3156 (15.6)	2431 (42.7)	< 10^–3^
CCB (%)	8028 (31.0)	4392 (31)	3630 (31)	0.9	6306 (31.2)	1722 (30.2)	0.14
Diuretics (%)	5514 (21.3)	3061 (21.6)	2446 (20.9)	0.17	4304 (21.3)	1210 (21.2)	0.8
Betablockers (%)	5734 (22.1)	3172 (22.4)	2554 (21.8)	0.27	4445 (22)	1289 (22.6)	0.3
Salt restriction (%)	13,891 (53.7)	7837 (55.3)	6043 (51.6)	< 10^–3^	10,789 (53.4)	3102 (54.4)	0.1
ABPM (%)	656 (2.5)	352 (2.5)	304 (2.6)	0.5	314 (1.6)	342 (6)	< 10^–3^
SMBP (%)	1630 (6.3)	868 (6.1)	762 (6.5)	0.2	1053 (4.1)	577 (10.1)	< 10^–3^
Drug compliance (%)	19,539 (75.5)	10,874 (76.8)	8651 (73.9)	< 10^–3^	15,326 (75.9)	4213 (73.9)	0.002
Physical activity (%)	3845 (14.9)	1598 (11.3)	2243 (19.2)	< 10^–3^	2748 (13.6)	1097 (19.3)	< 10^–3^

*Mann–Whitney nonparametric test

ABPM: Ambulatory blood pressure monitoring, ACE: Antagonist of conversion enzyme, ARB: Angiotensin Receptor Blockers, BMI: Body mass Index, CCB: Calcium Channels Blockers, COPD: Chronic obstructive pulmonary disease, DBP: Diastolic Blood Pressure, EKG: electrocardiogram, HTN: hypertension, LVH: Left Ventricle Hypertrophy, SD: Standard Deviation, SBP: systolic Blood Pressure, SMBP: Self measured Blood Pressure, TTE: Transthoracic echocardiography, yo: year-old

The mean age of our population was 64.0 ± 12.3 years (extremes: 18–108 years); half of our patients (49.3%) were more than 65 years old. There was a female predominance (sex ratio = 1.22). About 21.3% of the population was illiterate and only 9.4% got a university education. The rate of illiteracy was higher in women than in men (30.1% in females versus 10.8% in males). Most of the models had national health insurance (71.3%), private insurance was more frequently noted in patients treated in the private sector (5.8% versus 2.1%). The most associated risk factors were diabetes in 39.4% and obesity in 25.8%. Overweight or obesity was noted in 70.4%. The mean BMI of the study population was 28.1 ± 4.57, it was significantly higher in women than in men (p < 0.001), and obesity was more prevalent in female patients than in males (34.8% versus 17.6%). One patient out of four had a history of cardiovascular events (coronary disease or/and strokes), which means a very high cardiovascular risk.

The mean SBP during the office visit was 138.8 ± 19.6 mmHg and the mean DBP was 79.03 ± 11.1 mmHg. Using the cut-point of 140/90 mmHg as a BP target, 51.8% of our patients had a controlled BP, but using the lower cut-off recommended by the ACC/AHA of 130/80 mmHg, only 18.6% were controlled. Both DBP and SBP were significantly higher in men compared to women, but there were no significant differences between the private and the public sectors. Women were also more likely to present atrial fibrillation (8% versus 6.7%, p < 0.001) and hypothyroidism (8% versus 2.1%, p < 0.001). Female patients were significantly more controlled than males (52.3% versus 50.9%, p = 0.032). Generally, the male gender was found to be an independent predictor of uncontrolled hypertension. In fact, women were significantly more compliant to drug intake (76.6% versus 73.9%, p < 10^–3^) and to salt restriction (55.3% versus 51.6%, p < 10^–3^). Contrarily, men performed physical activities more frequently (p < 10–3).

The patients who were treated in the public sector were more frequently smokers (15% versus 10.7%, p < 10^–3^) and diabetics (40.5% versus 35.6%, p < 10^–3^) compared to those treated in the private sector, but they performed less BP out office measurement. Moreover, they had less frequent physical activities (13.6% versus 19.3%, p < 10^–3^). Thus, the rate of BP control was better in the private sector (57% versus 50.3%, p < 10^–3^ when we consider 140/90 mmHg as target).

The mean number of prescribed drugs was 1.49 ± 0.6, but 46.5% were on monotherapy and only 37.8% received two antihypertensive treatments or more. The percentage of multiple drugs (2 or more) was higher in the private sector (42.9% versus 36.5%). An Antagonist of Conversion Enzyme (ACE) or Angiotensin Receptor Blockers (ARB) were used in two patients out of three. The ACE class was more frequently prescribed in the public sector whereas the ARB class was more frequently used in the private sector.

On univariate analysis, patients with uncontrolled hypertension were significantly older, more frequently diabetic, obese, and smokers. They were followed in the public sector with a more frequent history of strokes and moderate renal failure antecedents. These patients also had a higher pulse rate (74.9 ± 11.4 bpm versus 72.5 ± 10.5 bpm, p =  < 0.001). They practiced sports less frequently (11.5% versus 18.1%, p < 0.001) and are less compliant to the drug intake and the salt restriction.

Control of BP was better during the holy month (Ramadan), among 15,239 hypertensive patients included during this month, with HTN history > 6 months, 53.3% of individuals were on target.

Contrariwise, patients with controlled hypertension had more frequently a history of coronary diseases than others (19.9% versus 18.8%, p = 0.033), underwent more frequently electrocardiogram and echocardiography control, and showed less frequently left ventricle hypertrophy (Table 2).

**Table 2:** Comparison of patients with controlled blood pressure to those with uncontrolled blood Pressure, based on univariate analysis

	Patients with Known hypertension > 6 months	Controlled BP (< 140/90)	Uncontrolled BP (≥ 140/90 mmHg)	p
N	23,601	12,206	11,395	
*Age (years)*				
Median ( min–max) Mean ± SD	65(18–118) 65.07 ± 11.9	65(18–118) 64.94 ± 12.15	65(18–104) 65.2 ± 11.6	**0**.022*
Male (%) Female (%) Sex Ratio (F/M)	10,543(44.7) 13,058 (55.3) 1.23	5371(50.9) 6835 (52.34) 1.23	5172(49.1) 6223 (47.6) 1.27	**0.032**
Public sector (%)	18,613 (78.9)	9361(76.7)	9252 (81.2)	< 10^–3^
Ramadan inclusion (%)	15,239 (64.6)	8132(66.6)	7107 (62.4)	< 10^–3^
*Education level*				
Unspecified (%) Illiterate (%) Primary school Secondary school University school	7182(30.4) 5236(22.2) 5111(21.7) 4041(17.1) 2031(8.6)	3688(30.2) 2588(21.2) 2548(20.9) 2217(18.2) 1165(9.5)	3494(30.7) 2648(23.2) 2563(22.5) 1824(16) 866(7.6)	< 10^–3^
Secondary/University education	6072(25.7)	3382(27.7)	2690(23.6)	< 10^–3^
*Health insurance coverage*				
Private insurance National insurance Indigent None Unspecified	624(2.6) 17,021(72.1) 4746(20.1) 620(2.6) 590(2.5)	331(2.7) 9012(73.8) 2275(18.6) 296(2.4) 292(2.4)	293(2.6) 8009(70.3) 2471(21.7) 324(2.8) 298(2.6)	< 10^–3^
*History of HTN*				
< 1 y (%) 1 to 5 y (%) 5 to 10 y (%) 10 to 15 y (%) > 15 y (%)	1240 (5.3) 5922 (25.1) 6293 (26.7) 4620 (19.6) 3187 (13.5)	177 (3.7) 1 138 (24.0) 1 434 (30.3) 1 486 (31.4) 503 (10.6)	690 (5.7) 3280 (26.9) 3225 (26.4) 2261 (18.5) 148 (12.1)	< 10^–3^
Smoking (%)	3226(13.7)	1525(12.5)	1701(14.9)	< 10^–3^
Diabetes (%)	9730(41.2)	4738(38.8)	4992(43.8)	< 10^–3^
Obesity (%)	6475(28.9)	3178 (27.4)	3297 (30.5)	< 10^–3^
*BMI*				
Median (min–max) Mean ± SD	27.5(13–72) 28.1 ± 4.5	27.4(13.7–67) 27.9 ± 4.5	27.6(14.6–72) 28.4 ± 4.6	< 10^–3*^
Moderate Renal Failure (%)	1479(6.3)	692(5.7)	787(6.9)	< 10^–3^
Hypothyroidism (%)	1289(5.5)	679(5.6)	610(5.4)	0.4
Confirmed Apnea syndrome (%)	443(1.9)	201(1.6)	242(2.1)	0.004
COPD (%)	502(2.1)	248(2)	254(2.2)	0.3
Stroke histoty (%)	1608(6.8)	765(6.3)	843(7.4)	< 10^–3^
Coronary disease (%)	4579(19.4)	2433(19.9)	2146(18.8)	0.033
Median Heart rate Mean Heart rate ± SD (bpm)	73(40–150) 73.6 ± 11	71(40–150) 72.4 ± 10.5	75(40–150) 74.8 ± 11.3	< 10^–3*^
Heart rate > 80 bpm (%)	4328(18.3)	1773(14.6)	2555(22.6)	< 10^–3^
LVH on EKG or TTE (%)	2728(11.6)	1213(9.9)	1515(13.3)	< 10^–3^
Atrial fibrillation (%)	1834(7.8)	970(7.9)	864(7.6)	0.3
Recent lab tests < 6 months	13,799(58.5)	7331(60.1)	6468(56.8)	< 10^–3^
Fasting Glucose (mg/dl)				
Median (min–max) Mean ± SD)	1.1(0.5–3.9) 1.3 ± 0.5	1.1(0.5–3.9) 1.6 ± 4.4	1.2(0.5–3.9) 1.9 ± 6.1	< 10^–3*^
Creatinine (µmol/l) Median ( min–max) Mean ± SD)	82(30–700) 93.07 ± 51.3	80(30–700) 90.7 ± 47.3	83(32–681) 95.5 ± 55.2	< 10^–3*^
Creatinine clearance (ml/min)				
Median ( min–max) Mean ± SD)	87.9(7–287) 89.9 ± 33.5	89(7.34–287) 91.2 ± 32	86.5(7.8–264) 88 ± 34	< 10^–3*^
*Total Cholesterol (mg/dl)*				
Median ( min–max) Mean ± SD)	1.8(1–5) 1.9 ± 0.6	1.8(1–5) 1.8 ± 0.6	1.8(1–5) 1.93 ± 0.6	0.01*
K + (mmol/l)				
Median (min–max) Mean ± SD)	4.1(2.4–7) 4.1 ± 0.46	4.1(2–7) 4.1 ± 0.45	4.1(2.5–7) 4.1 ± 0.5	0.3*
Proteinuria ( +)	1306(5.5)	616(10.7)	690(13.3)	< 10^–3^
*Drug treatment*				
No drug (%) Monotherapy (%) Bitherapy (%) Tritherapy or more (%)	3202(13.6) 10,959(46.4) 6318(26.8) 3122(13.2)	1583(13) 6294(51.6) 3131(25.7) 1198(9.8)	1619(14.2) 4665(40.9) 3187(28.0) 1924(16.9)	< 10^–3^
*Hypertensive drug*				
ACE (%)	10,734(45.5)	5602(45.9)	5132(45)	0.18
ARB (%)	5265(22.3)	3033(24.8)	2232(19.6)	< 10^–3^
CCB (%)	7324(31)	3739(30.6)	3585(31.5)	0.16
Diuretics (%)	5018(21.3)	2643(21.7)	2375(20.8)	0.12
Betablockers (%)	5239(22.2)	2726(22.3)	2513(22.1)	0.6
One single pill therapy (%)	1506 (6.3)	766 (3.2)	740 (3.4)	0.829
Salt restriction (%)	13,294(56.3)	8189(67.1)	5105(44.8)	< 10^–3^
ABPM (%)	493(2.1)	231(1.9)	262(2.3)	0.02
SMBP (%)	1464(6.2)	758(6.2)	706(6.2)	0.9
Drug compliance (%)	18,824(79.8)	10,566(86.6)	8258(72.5)	< 10^–3^
Physical activity (%)	3524(14.9)	2210(18.1)	1314(11.5)	< 10^–3^

*Mann–Whitney nonparametric test

**Fig. 1:**
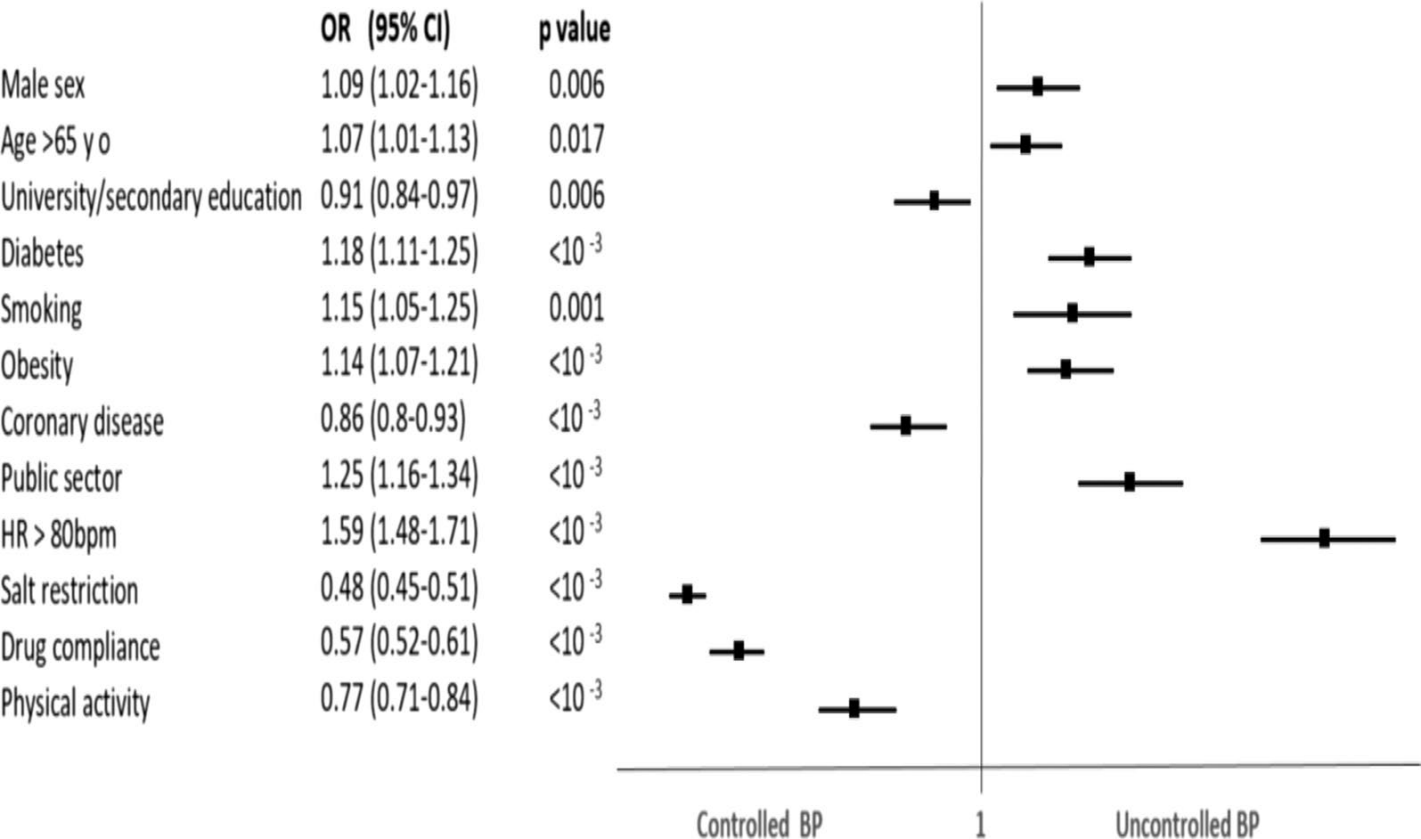
Forest Plot Graph: Predictors of blood pressure control according to the multivariate regression analysis. BP: Blood Pressure, bpm: beat per minute, CI: confidence Interval, HR: Heart rate, OR: odds ratio, Yo: year old

ABPM: Ambulatory blood pressure monitoring, ACE: Antagonist of conversion enzyme, ARB: Angiotensin Receptor Blockers, BMI: Body mass Index, CCB: Calcium Channels Blockers, COPD: Chronic obstructive pulmonary disease, DBP: Diastolic Blood Pressure, EKG: electrocardiogram, HTN: hypertension, LVH: Left Ventricle Hypertrophy, SD: Standard Deviation, SBP: systolic Blood Pressure, SMBP: Self measured Blood Pressure, TTE: Transthoracic echocardiography, Yo: year-old,

Based on multivariate analysis, predictors of uncontrolled hypertension were male gender, old age > 65 yo, diabetes, obesity, smoking, management in the public sector, and HR more than 80 bpm. Contrariwise, predictors of controlled hypertension were of high educational level (secondary/university), with a history of coronary disease, salt restriction, drug compliance, and regular physical activity (Table 3 and Fig. 1).

**Table 3:** Multivariate analysis: Independent predictors of Uncontrolled Blood Pressure

	OR	95% CI	P value
Male sex	1.09	1.02–1.16	0.006
Age > 65 y o	1.07	1.01–1.13	0.017
University or secondary education	0.909	0.84–0.97	0.006
Diabetes	1.18	1.11–1.25	< 10 ^−3^
Smoking	1.15	1.05–1.25	0.001
Obesity	1.14	1.075–1.219	< 10 ^−3^
Coronary disease	0.86	0.8–0.93	< 10 ^−3^
Public sector	1.25	1.16–1.34	< 10 ^−3^
HR > 80 bpm	1.59	1.48–1.71	< 10 ^−3^
Salt restriction	0.48	0.45–0.51	< 10 ^−3^
Drug compliance	0.57	0.52–0.61	< 10 ^−3^
Physical Activity	0.77	0.71–0.84	< 10 ^−3^

Bpm: beat per minute, CI: Confidence Interval, HR: Heart Rate, OR: odds Ration, Yo: year-old

## Discussion

Hypertension is the most prevalent cardiovascular factor worldwide and is the main cause of death even in developed countries [20]. Recently, a large meta-analysis of 2939 sudden cardiac deaths (SCD) among 418,235 participants from 18 studies showed that hypertension is associated with a twofold increase in the risk of SCD and a 28% increase of SCD per 20 mmHg increment in SBP [21]. Moreover, in a pooled dataset from 44 low-income and middle-income countries including 1,100,507 participants, the authors showed that only 10.3% of hypertensive patients achieved BP control [12]. All these indicators demonstrate that hypertension is a public health problem in developed as well as developing countries. Tunisia, is a middle-income country, and during the last decade, the Tunisian lifestyle, eating habits, and population aging have widely changed; there has been an increase in cardiovascular risk factors [22]. Recently, a national cross-sectional Tunisian study, “ATERA”, including 11,955 individuals showed that the prevalence of high blood pressure has increased to 50%, that of diabetes to 18%, and that of obesity to 31% [23]. Face to these dramatic epidemiologic indicators, the Tunisian Society of Cardiology and Cardiovascular surgery aimed to evaluate the cardiovascular profile of hypertensive patients and to assess the BP control, through a national flash study. To the best of our knowledge, NaTuRe HTN is the largest national survey of hypertension in Africa. The most important finding of this registry is that the profile risk of the Tunisians has changed remarkably. In 2012, Ben Romdhane et al. published the results of the TAHINA project, which was a Tunisian national survey including 8007 patients, aged between 35 and 70 years and examined across home visits. When comparing the results of TAHINA and NaTuRe HTN among hypertensive patients, we found that the prevalence of illiterate people has decreased from 43% to 21.3%. Surprisingly, we found that the prevalence of diabetes among hypertensive people has decreased from 62% to 39.4%, that of tobacco from 22 to 14%, and that of obesity from 46 to 25% [14]. All these findings demonstrate that, nowadays, the diagnosis of hypertension was made early before the development of diabetes and other comorbidities. That’s why the rate of diabetes among hypertensive patients has decreased. This finding goes along with the improvement of the educational level between the two studies; obviously, the Tunisian citizens’ awareness of blood pressure risks and management methods has increased.

The second important finding of our registry is the improvement of BP control. In 2005, Ben Romdhane et al. conducted a Tunisian cross-sectional survey on 1837 adults aged between 40–69 years old, only 13.2% of hypertensive individuals were controlled [11]. Hypertension control increased to 24.1% in the TAHINA project (2012), and recently we demonstrated in NaTuRe HTN that BP was controlled in 51.9% of our population when we consider 140/90 as a target. This rate is close to the rate achieved in many developed countries. Control of hypertension remains elusive nationally, despite the widespread availability of effective therapies.

In fact, controlling hypertension remains a health problem in not only low- and middle-income countries but also in high-income countries. Ikeda et al., in a comparative analysis of national surveys in 20 countries, showed that hypertension was treated in 13.8% to 80.5% of hypertensive patients in the different countries but was controlled only in 4.4% to 59.1% [13]. Recently, Pan et al. reported control of 60% of hypertensive patients in Taiwan, but the prevalence of diabetes, obesity, and smoking in this cohort were lower compared to our population [24].

In California, the implementation of a large-scale hypertension program has been associated with a significant increase in hypertension control compared to the other cities of the US. The control rate increased from 55% in 2001 to 64% in 2009. Key elements of this program included a comprehensive hypertension registry, development and sharing of performance metrics, evidence-based guidelines, medical assistant visits for blood pressure measurement, and single-pill combination pharmacotherapy [25].

Recently the FLASH 2019 study, a national French study, has shown a rate of 54% of BP control [26]. This rate was stable in between the different Flash studies (2009–2019), therapeutic inertia was advanced to explain the lack of BP control improvement. The monotherapy kept downgrading in the different guidelines but Girerd et al. reported that the rate of monotherapy has increased according to the different FLASH studies, changing from 44% in 2009 to 55% in 2019, and they related the cause to the difficulties of drug reimbursement during the last years.

In our population, BP control has improved; the reimbursement of Stage II and III hypertension costs, as well as the availability of generic molecules and the improvement of the education level of the Tunisian population, has certainly contributed to this achievement. The proportion of patients treated with monotherapy is still high (46.5%). The most prescribed monotherapy was ACE inhibitors. In fact, ACE inhibitors are available in hospital nomenclature but not ARB. In our study, 39% of the patients were diabetics, and 80% were treated in the public sector.

Management of patients in the public sector was found to be an independent predictor of uncontrolled hypertension. Certainly, this could be explained in part by the discrepancy of drug availability between the two sectors, the quality of health insurance, and the lack of one single pill treatment in the public sector. However, it is worth noting that in our cohort we found that patients treated in the public sector seem to be at a higher cardiovascular risk with higher prevalence of obesity, diabetes, smoking with less frequent physical activity. All these factors were identified as predictors of uncontrolled hypertension in our population and were behind the bad control of BP in the public sector. Moreover, patients treated in the private sector underwent more frequent out-office measurements. They had a lower heart rate and better follow-up with more frequent lab tests. We also noted that ARB Class was more frequently prescribed in the private sector and this class is associated with better tolerance and persistence. In the public sector, the majority of patients take their drugs from the hospital. ARB class was not available in the public sector. All these findings should be considered by the health ministry to improve the conditions of management of hypertensive patients in the public sector where patients with the highest cardiovascular profile are treated.

The reimbursement of stage I hypertension costs by the national security fund is another point to discuss and which is missing both, in the private and public sectors. There is an urgent need for a comprehensive integrated population-based intervention program to improve the serious problem of hypertension in Tunisia.

Heart rate was another strong predictor of uncontrolled BP in our population and this could be related to the big prevalence of overweight and obesity as well as the low physical activity practice. One patient out of five has a heart rate > 80 bpm in our model. Recently, the ESC/ESH guidelines classified this clinical finding among the factors influencing cardiovascular risk [17, 27]. The NICE guidelines recommended downgrading beta-blockers use and limiting their use to specific settings [28]. Sympathetic activation could be involved in the physiopathology of hypertension in Tunisian people [29], but before adding a beta-blocker, we should advise patients to lose weight and to practice moderate physical activity for at least 30 min, 3 days a week. Therapeutic education should be highly considered and practicing sports to reduce BP levels is highly recommended. In developed countries, 60% of the population practice sport regularly [30], in our study only 14% performed physical activity. Recently, Sata Rosa et al. showed that an active lifestyle improves heart rate variability, reduces oxidative stress in hypertensive people, and improves BP control [31]. On the other hand, Beta-blockers should not be dismissed, and patients with high HR definitely need this therapeutic class.

Patients with a history of coronary disease were more on target in our model and ischemic cardiomyopathy was even identified as a predictor of controlled blood pressure. Many previous studies have confirmed these findings [10, 11, 13, 32]. In fact, patients with coronary disease are more compliant with their drugs and generally receive at least two classes (beta-blockers and ACE or ARB); moreover, they consult their doctors more frequently.

## Limitations of the study

The main limitation of our registry is the fact that it included only confirmed patients and did not aim to assess the prevalence of hypertension or the rate of undiagnosed and non-treated hypertensive patients. If all these groups had been considered, the control rate would be lower.

Another limitation is the definition of hypertension control which was based on office measurements. We did not perform out-office measurements to check the white coat high blood pressure effect, therefore, the rate of uncontrolled patients could be over-estimated in this registry.

Finally, this cross-sectional study did not evaluate the clinical follow-up and the impact of uncontrolled blood pressure on cardiovascular events.

## Conclusions

NaTuRe HTN is the largest national survey of hypertension in Tunisia. It would contribute to analyzing the burden of hypertension in a developing country, and highlight the important gaps in the treatment of hypertensive individuals. Certainly, it may help to guide the implementation of future interventions and to write national guidelines. The most important finding of this registry is that the control of hypertension has remarkably improved over the last years, despite the high cardiovascular risk of our population. Therapeutic education along with substantial support to and interest in the public sector are important preventive measures that can help improve public health in Tunisia.

## Data Availability

Data cannot be shared publicly because of privacy concerns. Indeed, data might reveal the identity and the location of participants included in the study. Data are available from the Tunisian Society of Cardiology and Cardiovascular Surgery Ethics Committee (contact via Résidence Les pergolas, Rue du Lac Huron Appartement 201, Berges du Lac—Tunisie, Email: secretaire.stcccv@gmail.com; Tel: (+216) 71 965 432) for researchers who meet the criteria for access to confidential data. There were no administrative permissions required to access the raw data.

## References

[1] NCD Risk Factor Collaboration (NCD-RisC). Worldwide trends in blood pressure from 1975 to 2015: a pooled analysis of 1479 population-based measurement studies with 19·1 million participants. Lancet 2017;389(10064):37–55. 10.1016/S0140-6736(16)31919-5 PMC5220163 27863813

[2] Kearney PM, Whelton M, Reynolds K, Muntner P, Whelton PK, He J (2005). Global burden of hypertension: analysis of worldwide data. Lancet.

[3] Chow CK, Teo KK, Rangarajan S, Islam S, Gupta R, Avezum A (2013). Prevalence, awareness, treatment, and control of hypertension in rural and urban communities in high-, middle-, and low-income countries. JAMA.

[4] Cifkova R, Fodor G, Wohlfahrt P (2016). Changes in hypertension prevalence, awareness, treatment, and control in high-, middle-, and low-income countries: an update. Curr Hypertens Rep.

[5] Sarki AM, Nduka CU, Stranges S, Kandala N-B, Uthman OA (2015). Prevalence of hypertension in low- and middle-income countries: a systematic review and meta-analysis. Medicine (Baltimore).

[6] Prenissl J, Manne-Goehler J, Jaacks LM, Prabhakaran D, Awasthi A, Bischops AC (2019). Hypertension screening, awareness, treatment, and control in India: A nationally representative cross-sectional study among individuals aged 15 to 49 years. PLoS Med.

[7] Tocci G, Nati G, Cricelli C, Parretti D, Lapi F, Ferrucci A (2017). Prevalence and control of hypertension in the general practice in Italy: updated analysis of a large database. J Hum Hypertens avr.

[8] De Feo M, Del Pinto R, Pagliacci S, Grassi D, Ferri C (2021). Italian society of hypertension and federfarma. Real-world hypertension prevalence, awareness, treatment, and control in adult diabetic individuals: an italian nationwide epidemiological survey. High Blood Press Cardiovasc Prev.

[9] NCD Risk Factor Collaboration (NCD-RisC). Long-term and recent trends in hypertension awareness, treatment, and control in 12 high-income countries: an analysis of 123 nationally representative surveys. Lancet. 24 août 2019;394(10199):639–51. 10.1016/S0140-6736(19)31145-6 PMC6717084 31327564

[10] Whelton PK, He J, Muntner P (2004). Prevalence, awareness, treatment and control of hypertension in North America, North Africa and Asia. J Hum Hypertens.

[11] Ben Romdhane H, Skhiri H, Bougatef S, Ennigrou S, Gharbi D, Chahed MK (2005). Hypertension prevalence, awareness, treatment and control: results from a community based survey. Tunis Med.

[12] Geldsetzer P, Manne-Goehler J, Marcus M-E, Ebert C, Zhumadilov Z, Wesseh CS (2019). The state of hypertension care in 44 low-income and middle-income countries: a cross-sectional study of nationally representative individual-level data from 1·1 million adults. Lancet.

[13] Ikeda N, Sapienza D, Guerrero R, Aekplakorn W, Naghavi M, Mokdad AH (2014). Control of hypertension with medication: a comparative analysis of national surveys in 20 countries. Bull World Health Organ.

[14] Ben Romdhane H, Ben Ali S, Skhiri H, Traissac P, Bougatef S, Maire B (2012). Hypertension among Tunisian adults: results of the TAHINA project. Hypertens Res.

[15] Piepoli MF, Hoes AW, Agewall S, Albus C, Brotons C, Catapano AL (2016). European Guidelines on cardiovascular disease prevention in clinical practiceThe Sixth Joint Task Force of the European Society of Cardiology and Other Societies on Cardiovascular Disease Prevention in Clinical Practice (constituted by representatives of 10 societies and by invited experts)Developed with the special contribution of the European Association for Cardiovascular Prevention & Rehabilitation (EACPR). Eur Heart J.

[16] Saeedi P, Petersohn I, Salpea P, Malanda B, Karuranga S, Unwin N (2019). Global and regional diabetes prevalence estimates for 2019 and projections for 2030 and 2045: results from the International Diabetes Federation Diabetes Atlas, 9th edition. Diabetes Res Clin Pract.

[17] Williams B, Mancia G, Spiering W, Agabiti Rosei E, Azizi M, Burnier M (2018). 2018 ESC/ESH Guidelines for the management of arterial hypertensionThe Task Force for the management of arterial hypertension of the European Society of Cardiology (ESC) and the European Society of Hypertension (ESH). Eur Heart J.

[18] Levey AS, Stevens LA, Schmid CH, Zhang YL, Castro AF, Feldman HI (2009). A new equation to estimate glomerular filtration rate. Ann Intern Med.

[19] NCD Risk Factor Collaboration (NCD-RisC). Worldwide trends in body-mass index, underweight, overweight, and obesity from 1975 to 2016: a pooled analysis of 2416 population-based measurement studies in 128·9 million children, adolescents, and adults. Lancet. 2017;390(10113):2627–42. 10.1016/S0140-6736(17)32129-3 PMC5735219 29029897

[20] Zeng Z, Chen J, Xiao C, Chen W (2020). A global view on prevalence of hypertension and human develop index. Ann Glob Health.

[21] Pan H, Hibino M, Kobeissi E, Aune D. Blood pressure, hypertension and the risk of sudden cardiac death: a systematic review and meta-analysis of cohort studies. Eur J Epidemiol. 10.1007/s10654-019-00593-4 PMC7250808 31875269

[22] Ben Ayed H, Ben Jemaa M, Trigui M, Ben Hmida M, Kassis M, Jedidi J (2019). Cardiovascular diseases in Southern Tunisia: current trends and future projections. Tunis Med.

[23] Jemaa R, Razgallah R, Ben Ghorbel I, Rais L, Kallel A (2020). Prevalence of cardiovascular risk factors in the Tunisian population: The ATERA-survey. Arch Cardiovasc Dis Suppl.

[24] Pan H-Y, Lin H-J, Chen W-J, Wang T-D (2020). Prevalence, Treatment, Control and Monitoring of Hypertension: A Nationwide Community-Based Survey in Taiwan, 2017. Acta Cardiol Sin juill.

[25] Jaffe MG, Lee GA, Young JD, Sidney S, Go AS (2013). Improved blood pressure control associated with a large-scale hypertension program. JAMA.

[26] Girerd Xavier. Prise en charge de l’HTA en France : rien ne va plus [Internet]. Medscape. [cité 8 août 2020]. Disponible sur. http://francais.medscape.com/voirarticle/3605539.

[27] Julius S, Palatini P, Kjeldsen SE, Zanchetti A, Weber MA, McInnes GT (2012). Usefulness of heart rate to predict cardiac events in treated patients with high-risk systemic hypertension. Am J Cardiol.

[28] Krause T, Lovibond K, Caulfield M, McCormack T, Williams B (2011). Management of hypertension: summary of NICE guidance. BMJ.

[29] Esler M, Lambert G, Esler D, Ika Sari C, Guo L, Jennings G (2020). Evaluation of elevated heart rate as a sympathetic nervous system biomarker in essential hypertension. J Hypertens.

[30] Salman A, Sellami M, Al-Mohannadi AS, Chun S (2019). The associations between mental well-being and adherence to physical activity guidelines in patients with cardiovascular disease: results from the scottish health survey. Int J Environ Res Public Health.

[31] Santa-Rosa FA, Shimojo GL, Dias DS, Viana A, Lanza FC, Irigoyen MC (2020). Impact of an active lifestyle on heart rate variability and oxidative stress markers in offspring of hypertensives. Sci Rep.

[32] Vedanthan R, Kamano JH, DeLong AK, Naanyu V, Binanay CA, Bloomfield GS (2019). Community health workers improve linkage to hypertension care in Western Kenya. J Am Coll Cardiol.

